# Motivational Hierarchy in the Chinese Brain: Primacy of the Individual Self, Relational Self, or Collective Self?

**DOI:** 10.3389/fpsyg.2016.00877

**Published:** 2016-06-13

**Authors:** Xiangru Zhu, Haiyan Wu, Suyong Yang, Ruolei Gu

**Affiliations:** ^1^Institute of Cognition and Behavior, Henan University, KaifengChina; ^2^Institute of Psychology, Chinese Academy of Sciences, BeijingChina; ^3^Department of Psychology, Shanghai University of Sport, ShanghaiChina

**Keywords:** self, motivation, decision making, event-related potential (ERP), feedback-related negativity (FRN)

## Abstract

According to the three-tier hierarchy of motivational potency in the self system, the self can be divided into individual self, relational self, and collective self, and individual self is at the top of the motivational hierarchy in Western culture. However, the motivational primacy of the individual self is challenged in Chinese culture, which raises the question about whether the three-tier hierarchy of motivational potency in the self system can be differentiated in the collectivist brain. The present study recorded the event-related potentials (ERPs) to evaluate brain responses when participants gambled for individual self, for a close friend (relational self), or for the class (collective self). The ERP results showed that when outcome feedback was positive, gambling for individual self evoked a larger reward positivity compared with gambling for a friend or for the class, while there is no difference between the latter two conditions. In contrast, when outcome feedback was negative, no significant effect was found between conditions. The present findings provide direct electrophysiological evidence that individual self is at the top of the three-tier hierarchy of the motivational system in the collectivist brain, which supports the classical pancultural view that individual self has motivational primacy.

## Highlights

• A three-tier hierarchy of motivational potency exists in the self system.
• Evidence of the feedback-related negativity indicated that the individual self is at the top of the three-tier hierarchy in the collectivist brain.

## Introduction

The concept of the self occupies a central role in psychological theory, partly because of its relevance to cognitive, motivational, affective, and behavioral processes (Leary, 2007). The concept of the self is not a unitary phenomenon. Indeed, researchers have generally divided the self into individual self, relational self, and collective self (Greenwald and Pratkanis, 1984; Breckler and Greenwald, 1986; Triandis, 1989; Brewer and Gardner, 1996; Brewer and Chen, 2007). The individual self reflects cognitions that are related to traits, states, and behaviors that are stored in memory (e.g., “I am honest”). The relational self reflects cognitions that are related to one’s relationships (e.g., “I am a son”). The collective self reflects cognitions that are related to one’s groups (e.g., “I am Chinese”). The three kinds of selves are all necessary and are associated with psychological and physical health benefits. However, they are not equally important or meaningful. That is to say, one of them might be closer to the motivational core of the self-concept than the others. To provide a comprehensive understanding of the motivational hierarchy among the three kinds of selves, the present study evaluated the event-related potentials (ERPs) technique, combined with a gambling task to investigate the hierarchy of the self-motivation system in the collectivistic brain.

According to the three-tier hierarchy of motivational potency in the self-system, a series of experiments showed that the individual self is at the top of the motivational hierarchy, followed by the relational self and collective self (Sedikides et al., 2013). This idea has been confirmed by many studies (Gaertner et al., 1999, 2012). Gaertner et al. (2012) used the money allocation task and instructed the subjects to list goals for each self, they further employed groups of Chinese participants and found that the three-tier hierarchy applied to both Western (United States) and Eastern (Chinese) subjects. Consistent with this view, Abdukeram et al. (2015) used the method of the Twenty Statements Test and found the individual self is prominent compared with the relational self and collective self. These studies indicate that the primacy of the individual self is a universal phenomenon across cultural groups.

Nevertheless, some studies found that the motivational hierarchy systems are modulated by culture (Han et al., 2013; Kitayama and Park, 2014). Research on independent vs. interdependent self-construals is a prominent topic in social psychology. According to Markus and Kitayama (1991), the Western independent self is characterized as a self-contained and autonomous entity that is context independent and possesses salient internal attributes. The Eastern interdependent self, however, is treated as a member in a group and highlights personal belonging and dependence upon a context. Chinese self, but not the Western self, may include significant others. Indeed, other research revealed a different motivational hierarchy in Chinese people. For instance, by comparing the importance that Han ethnic groups placed on the three types of self, two studies found that relational self and private self in Han participants were ranked similarly, and both were more important than collective self (Huang et al., 2014; Mamat et al., 2014). The motivational hierarchy manifests itself not only in behavioral patterns but also in neural and electrocortical activities. Our previous study used a gambling paradigm and ERP technique. The feedback related negativity (FRN) results showed that the self and mother have the same motivational hierarchy in the Chinese brain (Zhu et al., 2015b). Another study found that friends also gain the same status in a self-motivation system (Kitayama and Park, 2014).

Given these inconsistent behavioral findings and the collectivist characteristics of Chinese culture, the role of the cultural factor deserves to be further explored when investigating motivational hierarchy in the Chinese brain. First, we aimed to explore whether friend has the same motivational hierarchy. According to Cai et al. (2013), the relational self can be subdivided into the familial self (involving family bonds) and the close other self (involving connections with a friend or romantic partner). Previous behavioral studies found that Chinese were closer to their parents, but friends were less important than their parents (Li, 2002; Cai et al., 2013). So we think that the status of friend is likely different from individual self and that of a family member. Second, previous behavioral studies found collective self is less important than relational self, but close other are confounded with family members in these studies. The present study aimed to compare the motivational hierarchy between close other and collective self.

The present study aims to explore potential electrocortical markers of the motivational hierarchy by examining the FRN. Feedback-related negativity is a key component of outcome evaluation, which is a medial frontal negative-going component that peaks approximately 250 ms following feedback presentation (Gehring and Willoughby, 2002). Localization studies suggest that the FRN is generated at the mPFC (Cohen et al., 2011). The FRN is an effective neural marker to explore the self motivational hierarchy because it is sensitive to the motivational factor. Specifically, the FRN amplitude is widely considered as an index of the motivational significance of the current event (Gehring and Willoughby, 2002; Yeung and Sanfey, 2004; Yeung et al., 2005; Leng and Zhou, 2010). In addition, the FRN reflects a semi-automatic outcome evaluation process which is immune to social desirability bias and test anxiety that might either exaggerate or obscure cultural differences. Hence, the present study adopted the FRN to investigate the self motivational hierarchy in Chinese college students.

The FRN has typically been viewed as a negative deflection in the ERP waveform that increases for monetary loss and is either reduced or absent for monetary gain (Holroyd and Coles, 2002). However, an accumulating body of recent evidence suggests the opposite viewpoint, in which the FRN amplitude is largely modulated by neural activity in gain trials (for a review, see Proudfit, 2015). One proposal is that monetary gain feedback elicits a distinct positive-going deflection (Holroyd et al., 2008; Baker and Holroyd, 2011). This reward positivity directly reflects activity of the mesencephalic dopamine system (Baker and Holroyd, 2011), a neural network that is critically involved in reward processing (Schultz, 2002). Reframing FRN as a response to monetary gain (i.e., a neurobiological index of hedonic capacity) makes it well-suited for studying the motivational hierarchy in the motivational system. Indeed, in the loss domain, there is little room to be “worse than expected” because losses are already the worst outcome. A previous study found that participants were more sensitive to the win condition than to the loss condition (Yu and Zhang, 2014). Pathological gamblers manifest insensitivity to losses but hypersensitivity to wins (Hewig et al., 2010). In another study, a group of depressed individuals presented blunted responses to gain feedback compared with the control group, whereas no significant group difference emerged for loss feedback (Liu et al., 2014). Based on these data, we predicted that the influence of the motivational hierarchy on FRN would be significant in the win domain (feedback related positivity or reward positivity) but not in the loss domain.

To sum up, the present study examined the motivational hierarchy among the individual self, close other, and collective self. We compared the FRN associated with outcome evaluation using a simple gambling task. In each trial, the beneficiary could be the individual self, relational self, or the collective self. Our hypothesis was that if the individual self, relational self, and collective self have different motivational hierarchies, then the FRN amplitude should reflect the hierarchical structure, such that a larger reward positivity indicates a higher motivational hierarchy.

## Materials and Methods

### Participants

Twenty one college students (all are Han people; 21.4 ± 0.8 years of age; range, 20–24 years; 10 females) participated in the study. Informed consent was obtained prior to the study. The experiment was conducted in accordance with the Declaration of Helsinki and was approved by the Ethics Committee of the Department of Psychology, Henan University, China. All of the participants had normal vision (with correction), and none had a history of neurological disease or brain injury. All of the participants were right-handed.

### Procedure

Before the simple gambling task, the participants selected a good friend (same sex but not romantic partner) to play for. In China, generally, dozens of students form a class, a class generally taking the same courses in 4 years. Each student affords a fixed amount money to establish the class fee. For the present study, participants come from different classes. Playing for class means that the money would be give to the class monitor and let all the class mates know this fact. The money should be used for class activities.

**FIGURE 1:**
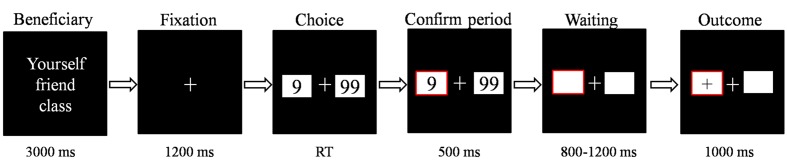
**The sequence of events within a single trial in the monetary gambling task.** In each trial, the beneficiary information lasted for 3000 ms then the fixation point lasted for 1200 ms. The participant was then presented with a choice of two alternatives, and the participant responded using the left or right index finger. The alternatives remained until the participant made his/her choice. Afterward, his/her choice was highlighted for 500 ms. After a subsequent interval of 8001200 ms, the participant received feedback, lasting 1000 ms, which indicated whether he/she gained or lost in that trial.

For the gambling task, the stimulus display and behavioral data acquisition were performed using E-Prime 1.1 software (Psychology Software Tools, Pittsburgh, PA, USA). During the task, the participants sat comfortably in an electrically shielded room approximately 80 cm from a computer screen. Each trial began with a 3000 ms presentation of the person for whom the participant was playing (i.e.,“for yourself,” “for your friend” and “for your class”). Two white rectangles (2.5°× 2.5° of visual angle) were then presented that contained two Arabic numerals (9 and 99) to indicate two alternative options on the left and right sides of a fixation point on the computer screen. The positions of the two numbers were counterbalanced across trials. The participants were asked to make a selection by pressing the “F” or “J” key on the keyboard with the left or right index finger, respectively. The alternatives remained on the screen until the participant chose one of the rectangles, which was then highlighted by a thick red outline for 500 ms. After a subsequent interval of 800–1200 ms, the participants received feedback, lasting 1000 ms, which indicated whether he/she gained (when the valence of the outcome was “+”) or lost (when the valence of the outcome was “-”) in that particular trial (see **Figure 1**). The formal task consisted of six blocks of 64 trials each. Unbeknownst to the participants, the outcomes were provided according to a predetermined pseudorandom sequence, and each participant received exactly 64 of each kind of outcome for each beneficiary. Each participant was paid 15 CNY for their participation in the study. In the gambling task, each beneficiary had 15 CNY in his/her account. Based on the points gained for each beneficiary, the final gain or loss was added to the separate account (every additional 500 points gained increase payment 5 CNY). The total payment for each participant was approximately 60.6 CNY (range, 4075 CNY; *SD* = 5.6 CNY).

Before the experiment, each participant was instructed about the rules and meaning of the symbols in the task. The participants were instructed that the money would be put on the friend’s cell phone or served as class fee. The participants were also encouraged to respond in such a way to maximize the total amount for each beneficiary. The participants were told that the higher the amount earned for each beneficiary, the more bonus money the beneficiary would receive at the end of the study. After the participant finished the task, he/she was told that the task had no optimal strategy.

### Electrophysiological Recording and Measures

Electroencephalographic (EEG) activity was recorded from 63 scalp sites using tin electrodes mounted in an elastic cap (Brain Products, Gilching, Germany) with an online reference to the middle at FCz at the standard locations according to the international 10–20 system and off-line re-referenced to the average reference. The horizontal electrooculogram (HEOG) was recorded from an electrode placed at the outer canthi of the right eye. The vertical electrooculogram (VEOG) was recorded from an electrode placed above the left eye. All inter-electrode impedance was maintained at <10 kΩ. The EEG and EOG signals were amplified with a bandpass filter from 0.05 to 100 Hz and continuously sampled at 500 Hz/channel.

Off-line analysis of the EEG was performed using Brain Vision Analyzer software (Brain Products). The first step in data preprocessing was the correction of ocular artifacts using Independent Component Analysis (ICA) of the continuous data using Brain Vision Analyzer 2.0 software. The ocular artifact-free EEG data were low-pass-filtered below 30 Hz (12 dB/oct) and high-pass-filtered above 0.1 Hz (12 dB/oct). Separate EEG epochs of 1000ms (200 ms baseline) were extracted oﬄine for the stimuli. All of the trials in which EEG voltages exceeded a threshold of ±75 μV during the recording epoch were excluded from the analysis (~7 trials per individual were excluded).

Through visual detection on the grand-averaged waveform, the FRN amplitude was measured for each participant as the average amplitude within the 220320 ms window (Boksem et al., 2012; Zhu et al., 2015a). The time window was extracted in a window extending 50 ms before and 50 ms after the peak latency. The electrodes at the mid-frontal region were selected for detecting the FRN (Frömer et al., 2016). Accordingly, the FRN amplitudes were entered into a 2 (feedback valence: win and loss) × 3 (beneficiary: individual self, friend and class) × 8 (electrodes: Fz, F1, F2, FC1, FC2, C1, C2, and Cz) repeated-measures analysis of variance (ANOVA).

## Results

### Behavioral Results

We defined the choice of ‘9’ to be the risk-avoidant choice in our experiment, predicting that participants would make this choice to avoid the possibility of a large loss (‘-99’). However, by making this choice, they also gave up the opportunity to receive the larger reward (‘+99’). In contrast, the choice of ‘99’ was defined as the risky choice (high-risk or high-return).

For the number of risky choice, the one-way repeated-measures ANOVA revealed no significant main effect of beneficiary (individual self, friend, and class), [*F*(2,40) = 2.44, *P* = 0.11, η*^2^* = 0.13]. For the RT (response time) data, the one way ANOVA revealed neither significant main effect nor and interaction effect, *P*s > 0.10.

#### ERP Results

**FIGURE 2:**
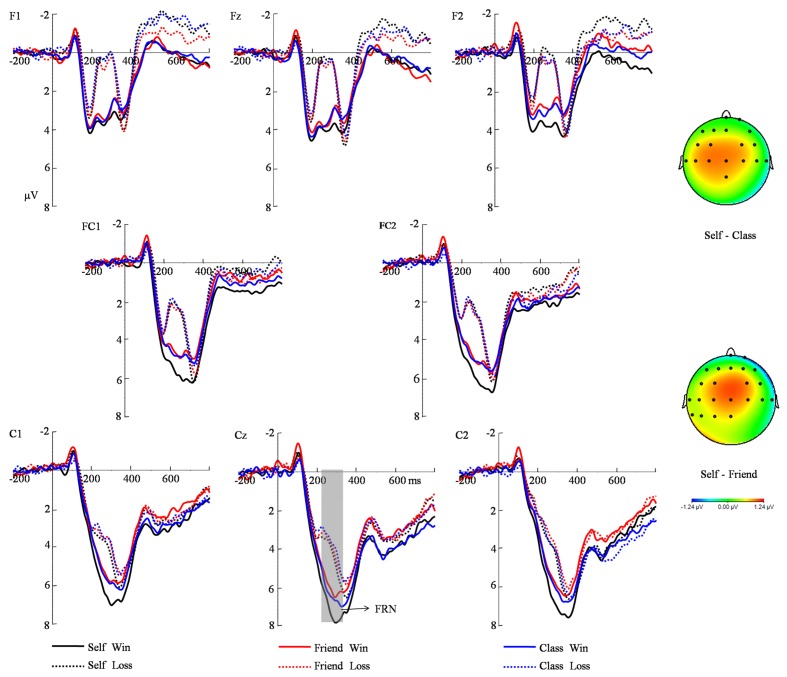
**Grand average FRN waveforms waves collapsed over reward magnitudes at eight electrodes post-onset of the feedback stimuli.** The topography maps indicate the FRN analysis window (220320 ms) for average amplitudes.

The main effect of feedback valence was significant [*F*(1,20) = 136.70, *P* < 0.001, η^2^ = 0.87], such that the FRN was more negative after losses (*M* = 2.09 μV, *SE* = 0.43) than after gains (*M* = 4.66 μV, *SE* = 0.54). The main effect of electrode on the FRN amplitude was also significant [*F*(7,140) = 22.89, *P* < 0.001, η^2^ = 0.53], with a largest amplitude at Cz site. The interaction between feedback valence and beneficiary was significant [*F*(2,40) = 4.09, *P* = 0.03, η^2^ = 0.17]. Simple effect analysis indicated that only in the win condition the effect of beneficiary was significant. Pairwise comparison revealed that winning for individual self (*M* = 5.40 μV, *SE* = 0.56) was larger than winning for friend (*M* = 4.23 μV, *SE* = 0.55) and winning for class (*M* = 4.36 μV, *SE* = 0.59) (*P* = 0.01, *P* = 0.009) (**Figure 2**). No significant difference existed between the latter two conditions. Neither the main effect of beneficiary nor other interactions were significant (all *Ps* > 0.05).

## Discussion

The present study investigated ERP responses to reward in a social context, in which the individual self, relational self, and collective self were the beneficiaries. Our main findings were threefold. First, behaviorally, no differences existed among the three kinds of selves. Second, the results replicated the well-established ERP patterns whereby win evoked larger reward positivity than loss in the gambling task. Third and most importantly, reward positivity was the largest when gambling for the individual self than for the relational or collective self, with no difference between the relational self and collective self. The present FRN results clearly support the pancultural view that the individual self is at the top of the motivational hierarchy.

The present results are consistent with the findings of previous studies (Gaertner et al., 2012; Abdukeram et al., 2015). Gaertner et al. (2012) reported that participants from China allocated more money to the individual self than to the relational self and collective self, indicating that the individual self was rated as most important in the self motivational system. Abdukeram et al. (2015) found relational aspect of an individual’s self became increasingly important with age in the Han cultural groups, but individual self still top the motivational hierarchy in 1024 years old participants.

However, the present results are inconsistent with Huang et al. (2014). In their study, participants were asked to write down five personal characteristics, five personal relationships, and five group memberships and then evaluate the importance they tie to each of them. As we pointed out in the introduction, the personal relationship may include family member and close others (friend or romantic partner). Given the important status of family member (Zhu et al., 2015b), it is likely to find no significant different between individual self and relational self.

Although Gaertner et al. (2012) proposed that the collective self is at the bottom of the motivational hierarchy, considerable uncertainty remains in the relative positioning of the relational and collective selves in Eastern cultures. One view posits that both selves rely on norms of interdependence, connectedness, and the importance of others and therefore might have equivalent motivational potency (Brewer and Chen, 2007). According to another view, collective behavior indicates that Eastern culture is more represented by interpersonal relationships that are internalized as the relational self than by in-group-associations that are internalized as the collective self, thus implying the relative primacy of the relational self (Yuki, 2003). In the present study, the relational self and collective self did not have different FRN. One potential reason is that friendship can be fleeting and depends largely on reciprocal exchange, therefore friend is not one of the key embeddedness in relational self. Another possible reason is that we used the participant’s class to represent the collective self. Participant may involve considerable dyadic relationships between the self and class, lead to the boundaries are not so obvious. Remaining unclear is whether differences between the relational self and collectivist self would become evident if we use a more abstract and important collective self.

In the present study, the motivational hierarchy of a friend was lower than the individual self. Notably, however, this motivational hierarchy is not absolute. Generally, the union with a close other, such as a friend, in Chinese culture is thought to be tight, and friends are also deeply ingrained in the self motivational system. For example, Kitayama and Park (2014) used error-related negativity (ERN) as a motivational neurological marker and found that it differentiated the self and friends in Western culture but not in East Asian culture. Two methodological differences that may account for this discrepancy. First, the beneficiary effect only manifested in the win condition but not in the loss condition, this result reflects dopaminergic signals response to positive outcomes (Baker and Holroyd, 2011), whereas ERN is thought to index the negative reward prediction errors that are based on a computation of an incorrect response as being worse than a correct response. Another reason is the speeded conflict task (flanker task) may be particularly likely to produce anxiety for Asians because this task is akin to an intelligence test. This anxiety may eliminate the difference between self and friend. Whereas the participants in the present study were presumed to feel safe while performing the gambling task (Hitokoto et al., 2016).

It should be noted that the self motivational hierarchy is not immune to the transient effect of temporal priming. For example, one previous recent fMRI study found that Chinese participants primed with independent self-construal showed stronger activations in the ventral striatum in response to winning money for the self than for a close friend, while those primed with interdependence self-construal showed comparable activations in two conditions (Varnum et al., 2014). This fMRI result indicates that self-construal could shapes self motivational hierarchy in a highly dynamic fashion.

In the present study, the ERP results indicated that individual self is on top of the motivational hierarchy, but the behavioral results revealed no motivational hierarchy. To explain this discrepancy, it is worth noting that behavioral research on the motivational hierarchy, which provides most of what we know about the three-tier hierarchy, are not immune to social desirability bias, because respondents are tend to answer in a socially acceptable way (van de Mortel, 2008). This social desirability bias may threaten the validity of the behavioral measures of motivational hierarchy accordingly. In contrast, neural measurements may provide more insights than behavioral methods. For example, in the study of Wang et al. (2012), behavioral questionnaires showed that the intimacy level of self-mother relationship and that of self-father relationship were not significantly different, but different neural representations of mother and father in the medial prefrontal cortex (mPFC) have been observed. Future studies that recruit alternative behavioral measures and neural markers should be conducted to examine our hypothesis.

One limitation is that we only included Han people in the present study. Although Chinese culture has been characterized as an interdependent culture, it has a certain degree of heterogeneity. Three recent studies considered intra-cultural variability in the self motivational hierarchy in China (Huang et al., 2014; Mamat et al., 2014; Abdukeram et al., 2015). Mamat et al. (2014) found that Uyghur Chinese rated the collective self as more important than the individual self and relational self. This was likely because the Uyghur culture is based on Islam, which emphasizes the solidarity of all Muslims. Their shared religion facilitates group integration, unity, and cohesiveness within the Uyghur ethnic group (Abdukeram et al., 2015). Future research that is devoted to exploring the motivational hierarchy should consider the intra-cultural variability of interdependent self-construal in Chinese populations. Another limitation is that we only employed Chinese participants in the present study, it would be advantageous if future research compares Chinese with western cultures to further explore how culture factor modulates motivational hierarchy.

## Conclusion

The FRN response to losses and gains in the gambling task provided electrocortical evidence that the individual self is at the top of the self motivational hierarchy in the Chinese brain, which supports the pancultural view that the individual self is more important than close other and collective self in the human motivational system.

## Author Contributions

XZ and RG designed research; XZ performed research; XZ analyzed data; HW and SY contributed analytic tools; XZ wrote the paper.

## Conflict of Interest Statement

The authors declare that the research was conducted in the absence of any commercial or financial relationships that could be construed as a potential conflict of interest.
